# Intracystic papillary neoplasm with an associated mucinous adenocarcinoma arising in Rokitansky–Aschoff sinus of the gallbladder

**DOI:** 10.1186/s40792-016-0189-7

**Published:** 2016-06-18

**Authors:** Ryuichiro Sato, Toshinori Ando, Hiroo Tateno, Toshiki Rikiyama, Toru Furukawa, Nobuo Ebina

**Affiliations:** Department of Surgery, Gonohe General Hospital, 17-3 Aza-Sawamukai, Sannohe-gun, Gonohe, 039-1517 Aomori Japan; Surgical Pathology Japan, Inc., Sendai, Japan; Department of Surgery, Saitama Medical Center, Jichi Medical University, Saitama, Japan; Tokyo Women’s Medical University Institute for Integrated Medical Sciences, Tokyo, Japan

## Abstract

Intraepithelial neoplasias are preinvasive neoplastic lesions found throughout in the digestive system, and when such lesions are discovered in the gallbladder, they are referred to as intracystic papillary neoplasm (ICPN). In the gallbladder, mucinous adenocarcinoma is a rare histologic phenotype, and adenocarcinomas involving Rokitansky–Aschoff (RA) sinuses are uncommon, which were indeed found in a case reported here. A 64-year-old male presenting with upper abdominal pain demonstrated a spherical mass protruding outward from the gallbladder fundus in imaging studies. Laparoscopic cholecystectomy was performed, and the resected specimen revealed a subserosal cystic mass with a small communication with the gallbladder lumen. The cystic mass contained a gelatinous material without solid component. Histologically, the mass was consisted of subserosal cysts lined by atypical columnar mucinous epithelium with micropapillary growth and nuclear stratification. The neoplastic transformation was more pronounced toward the serosal side of the lesion where disruption of the cyst wall, intrastromal mucin lakes, and invasion of the neoplastic cells into surrounding stroma were observed. The epithelium was of intestinal lineage, which was supported by the positive immunoreactivity against CDX2 and MUC2. The cystic spaces were communicated with surrounding RA sinuses, which indicated that the tumor arose in the sinus. The pathological diagnosis was ICPN, intestinal type, with an associated mucinous adenocarcinoma arising in RA sinus.

## Background

Intracystic papillary neoplasm (ICPN) of the gallbladder is a preinvasive neoplastic lesion and considered to share similar characteristics with intraductal papillary mucinous neoplasm (IPMN) and intraductal tubulopapillary neoplasm of the pancreas and intraductal papillary neoplasm of the extrahepatic bile duct. Mucinous adenocarcinoma of the gallbladder is a rare histologic phenotype, and adenocarcinomas involving Rokitansky–Aschoff sinuses (RA sinuses) are uncommon. Herein, we report a case exhibiting characteristics of the aforementioned three rare conditions, i.e., ICPN with an associated mucinous adenocarcinoma arising in RA sinus.

## Case presentation

A 64-year-old male presented with upper abdominal pain. He had suffered from similar abdominal pain for 5 months. The blood cell count and liver function tests were normal except elevated gamma-glutamyl transferase of 156 IU/L (normal range, 10–47 IU/L). Serum DUPAN-2 level (normal range, <150 U/ml) was modestly elevated to 296 U/ml, which was decreased to 64 U/ml after the surgery. CEA, CA 19-9, and SPan-1 levels were normal (2.0 ng/ml, 2.9 U/ml, and 10 U/ml, respectively).

**Fig. 1:**
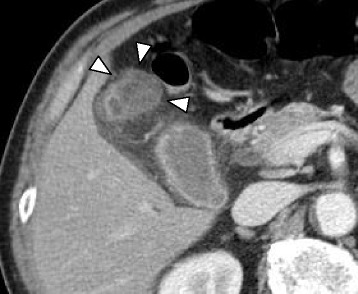
Computer tomography demonstrated a cystic mass protruding outward from the fundus of the gallbladder (*white triangles*). The tumor, 25 mm in diameter, showed mild contrast enhancement in the cyst wall on portal venous phase

**Fig. 2:**
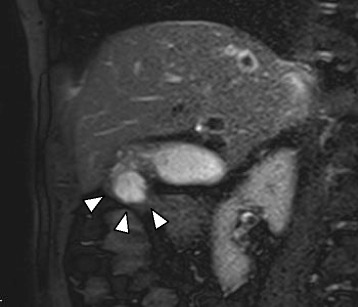
T2-weighted magnetic resonance imaging demonstrated a cystic mass at the gallbladder fundus (*white triangles*)

Computer tomography demonstrated a cystic mass protruding outward from the fundus of the gallbladder. The tumor, 25 mm in diameter, showed mild contrast enhancement in the cyst wall on portal venous phase (Fig. 1). Ultrasonography confirmed the low-echoic spherical mass. T2-weighted magnetic resonance imaging showed a cystic mass at the gallbladder fundus (Fig. 2). There was no stone nor anomaly in the extrahepatic bile duct, and high signal was not detected with diffusion-weighted image. Intramural nodule was not evident in any of the imaging studies performed.

**Fig. 3:**
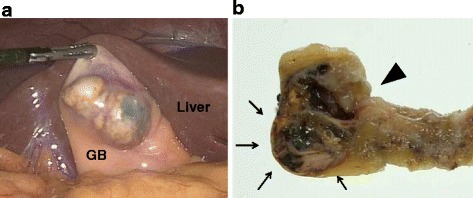
**a** A cystic mass was located at the serosal side of the gallbladder fundus. There was no sign of tumor invasion around the lesion. *GB* gallbladder. **b** Resected specimen revealed a mucin-filled subserosal cystic mass (*black arrows*) with a small communication with the gallbladder lumen (*black triangle*) at the gallbladder fundus. The cyst wall was partly thickened, which was proved to be severe fibrosis in histological evaluation. The remaining part of the gallbladder had thickened wall with denuded epithelium, consistent with chronic cholecystitis

Laparoscopic cholecystectomy was performed with a preoperative diagnosis of cystic gallbladder tumor. A cystic mass was located at the serosal side of the gallbladder fundus. No invasion into surrounding tissues was observed (Fig. 3). Postoperative course was uneventful.

**Fig. 4:**
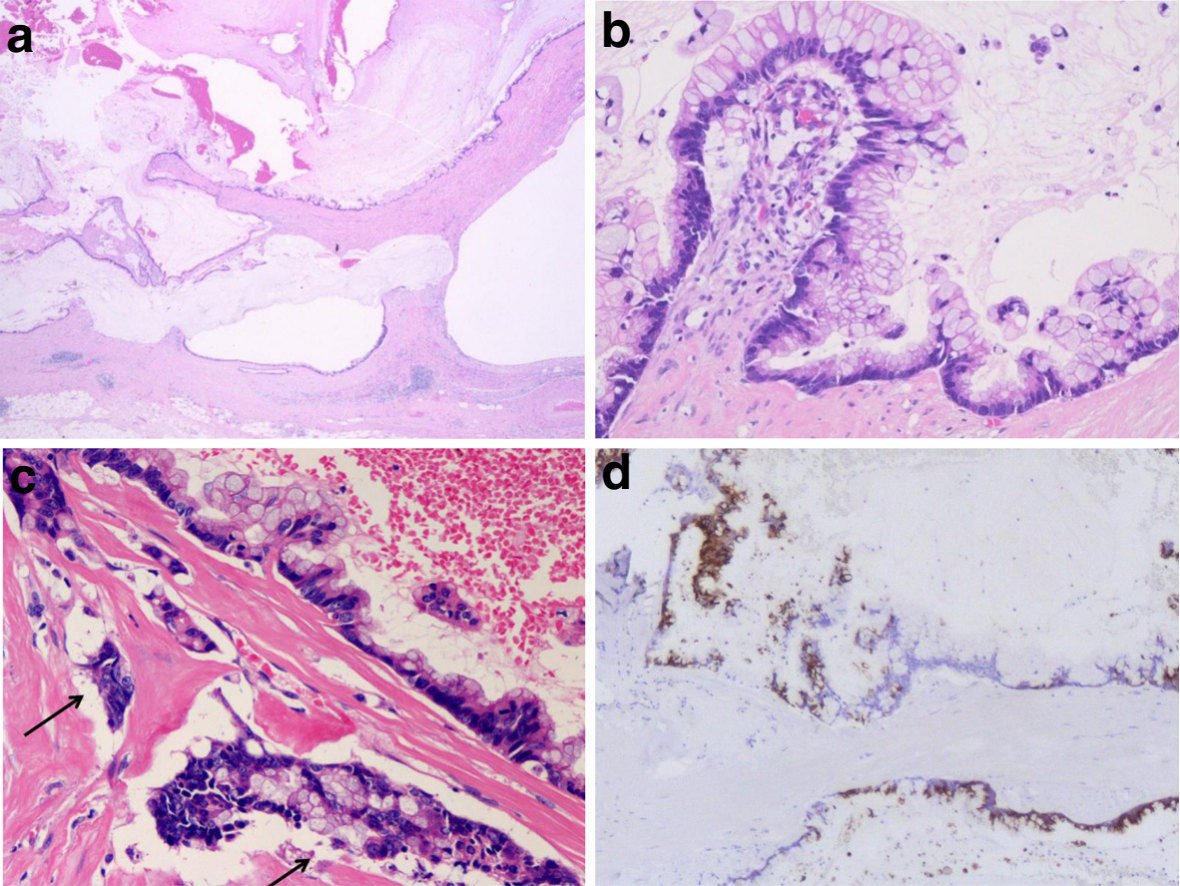
**a** The tumor consisted of subserosal multilocular cysts lined with columnar mucinous epithelium (hematoxylin and eosin). **b** The epithelium contained abundant intracytoplamic mucin and showed atypia with micropapillary growth and nuclear stratification with loss of polarity, which indicates neoplastic growth. **c** Stromal invasion of disorganized neoplastic cells were observed (*black arrows*). **d** The immunoreactivity against MUC2 was detected in the cytoplasm of the neoplastic epithelium

Pathological evaluation of the resected specimen revealed a mucin-filled subserosal cystic mass with a small communication with the gallbladder lumen at the gallbladder fundus. The cyst wall was partly thickened, which was proved to be severe fibrosis in histological evaluation. The remaining part of the gallbladder had thickened wall with denuded epithelium, consistent with chronic cholecystitis. Histologically, the tumor consisted of subserosal multilobular cysts lined by columnar mucinous epithelium (Fig. 4a). There were atypical foci with micropapillary growth and nuclear stratification (Fig. 4b). Dysplastic changes were present in varying degrees, from low- to high-grade dysplasia, and the latter was characterized by disorganized mucinous cells with mitotic figures, nuclear enlargement, and loss of polarity. The dysplastic changes were more pronounced at the serosal side of the cyst where disruption of the cyst wall, intrastromal mucin lakes, and stromal invasion of the neoplastic cells were observed (Fig. 4c). Signet-ring-like cells were seen floating within the mucin lake. The stroma in the lesion was highly fibrotic. Lymphovascular invasion, perineural invasion, and regional lymph node metastasis were not identified, and surgical margins were free of tumor. The lesion was communicated with RA sinuses that were abundantly found throughout the gallbladder wall, which indicated that the neoplasm arose in the sinus.

Immunohistochemical analysis revealed the neoplastic cells were positive for CK7, CDX2, MUC2 (Fig. 4d), and MUC5AC, while negative for CK20, MUC1, and MUC6.

The pathological diagnosis was ICPN, intestinal type, with an associated mucinous adenocarcinoma arising in RA sinus.

### Discussion

Intraepithelial neoplasias are preinvasive neoplastic lesions found throughout in the digestive system, and when such lesions are discovered in the gallbladder, they are referred to as ICPN [1, 2]. ICPN was reported to be found in 14 out of 3265 cholecystectomies (0.4 %). The frequency might be higher among invasive carcinomas, and 39 out of 606 (6.4 %) carcinoma cases had ICPN component. Conversely, invasive carcinoma was seen in 68 out of 123 (55 %) ICPN cases [2].

Mucinous adenocarcinoma is a rare histologic phenotype among gallbladder adenocarcinomas, and by conventional definition, more than 50 % of the tumor contains extracellular mucin [1]. Dursun et al. reported that among 606 gallbladder carcinomas, 40 cases (6.6 %) showed some degree of mucin production, and only 15 cases (2.5 %) were qualified as mucinous adenocarcinoma, with 3 cases associated with ICPN [3]. Majority of the invasive carcinomas arising from ICPN were pancreatobiliary-type adenocarcinomas, and only 4 out of 68 (5.9 %) cases were mucinous adenocarcinoma [2]. This was in contrast to IPMN of the pancreas, from which mucinous colloid-type adenocarcinomas were more commonly developed [4].

RA sinuses are the epithelial invaginations extending to the fibromuscular or subserosal layers through the gaps between the smooth muscle. Terada reported that RA sinuses were recognized in 351 out of 540 cholecystectomy specimens (65 %), and their epithelium occasionally showed marked proliferation [5]. Cases of gallbladder carcinoma arising in or extending toward the RA sinuses were reported, which were typically pancreatobiliary-type adenocarcinoma [6–9]. They pose a diagnostic challenge, since it is sometimes difficult to distinguish neoplastic cells extending into the RA sinuses from those with stromal invasion [6]. Albores-Saavedra et al. reported cases where RA sinuses with benign epithelium accompanied intrastomal mucin deposits [10] or pseudoperineural invasion [11], which could be misinterpreted as invasive carcinoma. Precise pathological evaluation of the specimen is vital since it affects the patient’s management including radical re-resection.

In the present case, the papillary epithelium with abundant extracellular mucin showed various degrees of cytoarchitectural atypia, from low- to high-grade dysplasia. There were disruption of the cyst wall, intrastromal mucin lakes with floating clusters of the neoplastic cells, and stromal invasion of the neoplastic cells, indicating that the neoplasm was invasive mucinous adenocarcinoma. The epithelium was of intestinal lineage, which was supported by the positive immunoreactivity against CDX2 and MUC2. The intestinal lineage is one of the five lineages comprising ICPN: biliary, gastric foveolar, gastric pyloric, intestinal, and oncocytic and account for 8 % of the cases [2]. CDX2 expresses mostly in small and large intestinal epithelium and plays an essential role in regulating proliferation and differentiation of the epithelium together with MUC2 [12]. CDX2 and MUC2 are fairly specific markers of intestinal differentiation and mucinous colloid-type carcinomas of the exocrine glands. In the gallbladder, MUC2 expression is not observed in the normal luminal epithelium and is exceedingly uncommon in pancreatobiliary-type adenocarcinoma [3]. The histological as well as immunohistochemical profiles in the present case resembled those of the IPMN of the pancreas, in particular, of the intestinal subtype. They were suggested to transform into mucinous colloid-type carcinomas through the intestinal pathway of carcinogenesis [4]. In the present case, the tumor lined with dysplastic epithelia was communicated with RA sinuses, indicating that the neoplasm arose in the RA sinus. To the best of our knowledge, there were only two cases of in situ carcinoma exhibiting similar characteristics as the present case reported in the literature [6, 13], and our case is the first case with invasive carcinoma.

The unusual presentation of our case had made it difficult to diagnose preoperatively. Besides malignancy, primary hydatid cyst, lymphangioma, and mucinous cystic neoplasm (MCN) are cystic lesions of the gallbladder reported in the literature [14–17]. MCN is a cystic neoplasm with low malignant potential composed of tall columnar cells with intracytoplasmic mucin. MCN is accompanied by ovarian-type stroma, densely packed spindle-shaped cells beneath the epithelium immunohistochemically positive for estrogen receptor, progesterone receptor, calretinin, and inhibin α, which is regarded as a prerequisite for diagnosis [1, 17]. In the present case, the ovarian-type stroma was absent, which was confirmed by negative immunohistochemical staining against those markers. DUPAN-2 level was modestly elevated to 296 U/ml, which is below the cutoff value of 400 U/ml advocated in the literature [18]. DUPAN-2 was reported to be elevated in pancreatic cancer, biliary tract cancer, and some benign diseases, especially obstructive jaundice [18, 19]. The DUPAN-2 level decreased after the surgery, suggesting that the value might be useful in early detection of the disease recurrence.

Interpreting the pathology report and reflecting it to the patient’s management required careful consideration. Cholecystectomy alone might not be an adequate therapy for gallbladder adenocarcinoma with subserosal invasion, since radical re-resection including hepatic resection and portal lymph node dissection resulted in a significant survival advantage [20]. Moreover, mucinous carcinoma was reported to have significantly worse overall survival than conventional adenocarcinoma [3]. The RA sinus involvement was related to adverse outcome among early gallbladder carcinomas [9]. On the other hand, prognosis of ICPNs with invasive carcinoma was far better than that of pancreatobiliary-type invasive carcinoma, with 3- and 5-year survival rate of 60 and 60 %, respectively [2]. CDX2 was reported to be an independent favorable prognostic factor in gallbladder carcinomas [12, 21]. In the present case, the small invasive foci found in a subserosal layer might be treated differently from the carcinoma invading from the epithelium to subserosa. Lymphovascular invasion, perineural invasion, and regional lymph node metastasis were not identified. Taken these findings into consideration, we did not perform adjuvant therapy and carefully followed up the patient who is disease free for 8 months after the surgery.

## Conclusions

In summary, this report presented a rare case of ICPN with an associated mucinous adenocarcinoma arising in RA sinus of the gallbladder, and this was the first reported case with invasive carcinoma. Surgeons and surgical pathologist should be familiar with the disease which requires meticulous clinicopathological evaluation and careful patient management.

## Consent

Written informed consent was obtained from the patient for the publication of this case report and any accompanying images. A copy of the written consent is available for review by the Editor-in-Chief of this journal.
